# Developing 3D microscopy with CLARITY on human brain tissue: Towards a tool for informing and validating MRI-based histology

**DOI:** 10.1016/j.neuroimage.2017.11.060

**Published:** 2018-11-15

**Authors:** Markus Morawski, Evgeniya Kirilina, Nico Scherf, Carsten Jäger, Katja Reimann, Robert Trampel, Filippos Gavriilidis, Stefan Geyer, Bernd Biedermann, Thomas Arendt, Nikolaus Weiskopf

**Affiliations:** a Paul Flechsig Institute of Brain Research, University of Leipzig, Liebigstr. 19, 04103, Leipzig, Germany; b Department of Neurophysics, Max Planck Institute for Human Cognitive and Brain Sciences, Stephanstraße 1a, 04103, Leipzig, Germany; c Center for Cognitive Neuroscience Berlin, Free University Berlin, Habelschwerdter Allee 45, 14195, Berlin, Germany

## Abstract

Recent breakthroughs in magnetic resonance imaging (MRI) enabled quantitative relaxometry and diffusion-weighted imaging with sub-millimeter resolution. Combined with biophysical models of MR contrast the emerging methods promise *in vivo* mapping of cyto- and myelo-architectonics, i.e., *in vivo* histology using MRI (hMRI) in humans. The hMRI methods require histological reference data for model building and validation. This is currently provided by MRI on post mortem human brain tissue in combination with classical histology on sections. However, this well established approach is limited to qualitative 2D information, while a systematic validation of hMRI requires quantitative 3D information on macroscopic voxels.

We present a promising histological method based on optical 3D imaging combined with a tissue clearing method, Clear Lipid-exchanged Acrylamide-hybridized Rigid Imaging compatible Tissue hYdrogel (CLARITY), adapted for hMRI validation. Adapting CLARITY to the needs of hMRI is challenging due to poor antibody penetration into large sample volumes and high opacity of aged post mortem human brain tissue. In a pilot experiment we achieved transparency of up to 8 mm-thick and immunohistochemical staining of up to 5 mm-thick post mortem brain tissue by a combination of active and passive clearing, prolonged clearing and staining times. We combined 3D optical imaging of the cleared samples with tailored image processing methods. We demonstrated the feasibility for quantification of neuron density, fiber orientation distribution and cell type classification within a volume with size similar to a typical MRI voxel. The presented combination of MRI, 3D optical microscopy and image processing is a promising tool for validation of MRI-based microstructure estimates.

## Highlights

• CLARITY on post mortem human brain was adapted for validation of MRI-based histology.
• Clearing of up to 8 mm, 3D imaging and staining of up to 5 mm thick samples was demonstrated.
• Multiple stains were applied including markers for fibers, neurons and glia.
• Characterization of cyto- and myelo-architectonics within an MRI-voxel was shown.

## Introduction

Recent technological breakthroughs translated magnetic resonance imaging (MRI) from a purely qualitative to a quantitative imaging technique, with resolutions in the sub-millimeter scale (van der Zwaag et al., 2016). Layer specific information on cortical function and intracortical connectivity is becoming available *in vivo* from ultra-high resolution structural, functional and diffusion-weighted imaging (DWI) (Bazin et al., 2014, Dinse et al., 2015, Leuze et al., 2014, Marques et al., 2017, Setsompop et al., 2013, Setsompop et al., 2017, Trampel et al., 2017, van der Zwaag et al., 2016). Parallel development of theoretical models linking cortical cyto- and myelo-architectonics at the micrometer scale with quantitative MR-parameters enables extraction of biologically relevant microstructural information from the whole brain MRI (Jespersen et al., 2010, Mohammadi et al., 2015, Stikov et al., 2015, Stüber et al., 2014, Zhang et al., 2012). Exploitation of quantitative imaging in conjunction with biophysical modeling opens an exciting route towards MRI-based *in vivo* histology (hMRI) (Weiskopf et al., 2015), such as *in vivo* measurements of cortical myelo-architectonics (Sereno et al., 2013) and *in vivo* Brodmann mapping (Dinse et al., 2015, Turner and Geyer, 2014). The recent achievements in DWI, such as an increased magnetic field and gradient strength (Heidemann et al., 2012, Setsompop et al., 2013, Sotiropoulos et al., 2013, Vu et al., 2015) and advanced biophysical contrast models (Novikov et al., 2016, Zhang et al., 2012), promise to enable the investigation of intracortical fibers with submillimeter resolution. If successful, this novel approach would be a powerful tool for studying cortico-cortical connectivity in humans, which is still largely unexplored.

The hMRI approach requires careful validation by independent methods. The combination of MRI and classical histology on post mortem tissue sections is currently the primary validation approach (Geyer and Turner, 2013). This combination allows for direct comparison of hMRI with histological ground truth. It also provides crucial reference data for model building. However, this approach suffers from significant limitations, since classical histology only provides qualitative 2D data instead of quantitative 3D information required for hMRI validation and biophysical modeling. In particular, validation of DWI-based methods estimating fiber orientation, densities, distributions and tracts cannot be reliably and systematically performed on 2D histological slices, since they do not allow estimating these metrics in an unbiased fashion (or at all). Thus, a comprehensive validation of intracortical and white matter fiber tracking is currently challenging when more than one main fiber direction has to be extracted (Leuze et al., 2014).

Several advanced histological methods were recently used to overcome these limitations. Polarized light imaging (PLI) (Axer et al., 2011, Axer et al., 2016, Leuze et al., 2014, Zilles et al., 2016), optical coherence tomography (OCT) (Magnain et al., 2014, Magnain et al., 2015, Wang et al., 2014), polarized sensitive OCT combined with block face imaging (Wang et al., 2016) provided valuable insights into cyto- and myeloarchitecture for validation of MR metrics (Leuze et al., 2014, Mollink et al., 2017). Particularly, 3D versions of PLI (Axer et al., 2016) and PS OCT (Wang et al., 2016) combined with block face imaging are very promising. However, these methods are not easily combined with flexible immuno-histochemical labeling. Therefore each of these methods provides an insight in one particular aspect of cortical microarchitectonics. Thus, additional tailored histological methods for validation of hMRI are highly needed.

Here, we explore the potential of 3D microscopic optical imaging and CLARITY tissue clearing (Chung and Deisseroth, 2013, Leuze et al., 2017) to address some of the above described limitations.

The first method to make animal and human tissue transparent for 3D investigations of organs and tissues was proposed by the Leipzig anatomist Werner Spalteholz (1914) more than hundred years ago. Recently his idea underwent a fascinating renaissance with CLARITY and other recent clearing approaches (Chung et al., 2013, Costantini et al., 2015, Liebmann et al., 2016, Liu et al., 2016, Phillips et al., 2016, Renier et al., 2014, Richardson and Lichtman, 2015). In particular, combined with new advances in microscopic technology it was used for 3D imaging at cellular and subcellular resolution (Dodt et al., 2007). The CLARITY tissue clearing approach consists of two main steps: 1) cross-linking of tissue macromolecules in hydrogel followed by 2) removal of lipids from the tissue (Chung and Deisseroth, 2013). After subsequent refractive index matching, blocks of tissue are optically highly transparent. Remarkably, CLARITY preserves the tissue's native protein composition and its fine structure. It can therefore be combined with multiple fluorescent immunohistochemical staining techniques and allows for histological imaging in 3D.

However, CLARITY has primarily been applied to tissue from young transgenic rodents, and its translation to large post mortem human brain tissue specimens faces a number of challenges (Liu et al., 2016). In particular, high opacity in mature post mortem human brain resulting from high myelin density, age related lipofuscin accumulation, protein aggregation and blood-residual auto-fluorescence of unperfused tissue are some of the main limitations (Liu et al., 2016). Most of the studies using CLARITY on post mortem human brain tissue were therefore performed on 100–1 000 μm thin slices (Ando et al., 2014, Chung et al., 2013, Chung et al., 2013, Costantini et al., 2015, Phillips et al., 2016), whose extent is insufficient for comparisons with hMRI and intracortical fiber tracking over distances longer then 500 μm. Processing of thicker samples is challenging, since both clearing and immunohistochemical staining are dependent on diffusion, which requires longer time in thick samples. Another challenge for the comparison of hMRI and CLARITY-based 3D microscopy is tissue distortion and expansion upon lipid loss, which make voxel-by-voxel comparisons between the modalities challenging.

This paper lays out the method and workflow to overcome some of these challenges. It reports on successful pilot studies adapting the active CLARITY method to mature post mortem human brain samples with a thickness of up to 8 mm. In the following we first describe an optimized clearing and immunohistochemical staining procedure combined with tailored image processing tools. We demonstrate feasibility of 3D microscopic imaging of cortical cyto- and myelo-architectonics covering the volume of a typical MR voxel. We further show, that using dedicated image analysis tools it is possible to access orientation distributions of intracortical fibers and 3D distribution of pyramidal and non-pyramidal cells within the cortex. We discuss limitations and methodological challenges due to remaining light scattering, CLARITY-related tissue distortion, limitations of currently available optical imaging systems and strategies to overcome these limitations.

## Methods

Clearing experiments were performed on large blocks of post mortem tissue described in detail in Section 2.1. Tissue blocks were cleared using optimized clearing procedure as described in Section 2.2. After blocks achieved high optical visual transparency (visual assessment) they underwent several experiments with the goal to answer the following questions:

1) Is the tissue protein content and tissue microstructure preserved by clearing?

To this end, one part of the cleared blocks was dissected into smaller subsamples, each stained with one or multiple antibodies (as described in Section 2.3) and imaged with fluorescence optical imaging described in Section 2.5.

2) Is the antibody penetration in the thick blocks sufficient for achieving effective immunohistochemical staining?

To address this question, three selected stains were performed on larger subsamples as described in Section 2.3 and imaged in 3D with optical imaging systems described in Section 2.5.

3) Is the tissue size and geometry preserved by clearing?

This question was addressed by monitoring the size and the shape of the samples with quantitative MRI as described in Section 2.4 at all clearing stages.

4) Does the optical transparency and staining efficiency allow for 3D optical imaging with sufficient quality to enable for automated studies of cyto- and myeloarchitectonics?

In order to answer this question, the obtained 3D optical imaging data were analyzed with tailored image processing tools described in Subsection 2.6 and 2.7, Subsection 2.6 and 2.7 targeting automated analysis of cyto- and myeloarchitectonics, respectively.

### Post mortem human brain tissue

Blocks from three human post mortem brains (Case 1: temporal lobe - female, 69 years old, post mortem interval prior to fixation (PMI) 24 h, two blocks with sizes about 25 mm × 35 mm x 8 mm and 30 mm × 50 mm x 8 mm; Case 2: temporal lobe - male, 54 years old, PMI 96 h, block size 30 mm × 38 mm x 8 mm; Case 3: corpus callosum - 70 years old, male, PMI 40 h, size 10 mm × 20 mm x 5 mm) were provided by the Brain Banking Centre Leipzig of the German Brain-Net (GZ 01GI9999-01GI0299), operated by the Paul Flechsig Institute of Brain Research (Approval # 82–02). The entire procedure of case recruitment, acquisition of the patient's personal data, the protocols and the informed consent forms, performing the autopsy, and handling the autopsy material have been approved by the responsible authorities. Following the standard Brain Bank procedures, blocks were immersion-fixed in 4% paraformaldehyde in phosphate buffered saline (PBS) pH 7.4 at least 6 weeks. Blocks were then stored in 30% sucrose in PBS with 0.1% sodium azide at 4 °C. Prior to the clearing procedure the blocks were washed in PBS for 7 days.

### Optimized CLARITY procedure

After washing in PBS, samples were incubated for 10 days in a hydrogel solution containing 4% paraformaldehyde (PFA), 2% acrylamide (Roth, Karlsruhe, Germany), 0.05% (vol/vol) bis-acrylamide (Roth, Karlsruhe, Germany) and 0.25% (wt/vol) VA-044 thermal initiator (Wako, Alpha-Labs, Eastleigh, UK) in PBS at 4 °C (modified from (Costantini et al., 2015)). Afterwards, the hydrogel solution with the tissue samples was saturated with nitrogen for 30 min, evacuated to 100 mbar and polymerized at 37 °C for 3 h. Excessive gel was thoroughly removed from the tissue. Hydrogel-embedded tissue was then washed at room temperature to remove lipids using sodium dodecyl sulphate (SDS) detergent clearing solution (4% SDS in 200 mM boric acid solution, pH 8.5). For the passive clearing samples were incubated at 37 °C in SDS clearing solution, which was replaced every 2–3 days.

After two weeks of passive clearing the tissue underwent active electrophoretic clearing (8 V, 1.2 A, 37 °C) for 2 weeks. For that, a 5-L vertical electrophoresis chamber (Biostep GmbH, Jahnsdorf, Germany) with an active temperature control (LTF UH4/14 D, Wasserburg, Germany) was modified for the special requirements of this process (adopting size of the chamber). Subsequently, the samples again underwent passive clearing at 37 °C for time periods ranging from 15 weeks (Case 3) to 14 month (Case 2 and part of Case 1). Total clearing times for each subsample are indicated in Table 1. The main difference of the procedure used in this study as compared to previously published approaches was the combination of passive and active clearing, mild clearing conditions and long clearing times (up to 14 months). Note, that the clearing was performed on larger tissue blocks with their original sizes.

**Table 1:** List of investigated subsamples.

Subsample	Case	Size mm x mm x mm	Clearing times	Stain (antibodies)	Microscope	Figures, Videos
1 A	1	3 × 3 x 5	10 month	neurons (HuC/D)	LaVision	Fig. 2, Fig. 5, Video S1
1 B	1	3 × 3 x 3	10 month	fibers (MBP)	Zeiss	Fig. 2b, Video S2
1C	1	3 × 3 x 3	10 month	interneurons (parvalbumin)	Zeiss	Fig. 2c
1D	1	3 × 3 x 3	10 month	neurons, fibers astroglia (HuC/D, MBP, GFAP)	Zeiss	Fig. 2d,e,f
1E	1	3 × 3 x 3	10 month	GFAP	Zeiss	Fig. 2g
1F	1	3 × 3 x 3	10 month	microglia (Iba-1)	Zeiss	Fig. 2h
1G	1	3 × 3 x 3	10 month	vessels (helix aspersa agglutinin)	Zeiss	Fig. 2i
1I	1	5 × 5 x 3	14 month	neurons, astroglia (HuC/D, GFAP)	Zeiss	Fig. 3
1H	1	20 × 20 x 8	10 month	fibers (MBP)	LaVision	Fig. 6, Fig. 7, Video S3, Video S4
2 A	2	5 × 5 x 5	14 month	neurons (HuC/D)	LaVision	Video S5
3 A	3	3 × 3 x 3	15 weeks	fibers (MBP & Tubulin)	Zeiss	Fig. 2k,l,m

### Immunostaining of clarified human brain tissue samples

The cleared blocks were washed thoroughly with PBS-Tween^20^ (PBS-T; 0.1% Tween^20^ and 0.01% sodium azide in PBS) for 24 h at room temperature to remove residual SDS micelles.

Smaller subsamples listed in Table 1 were dissected out of tissue blocks and used for following experiments.

To test protein content and microstructure of cleared tissue, parts of tissue blocks (Case 1 and 2 covering grey matter and Case 3 containing dense white matter) were cut into smaller subsamples (1 A-G and 3 A, see Table 1) and used for multiple fluorescent staining and 2D imaging. Subsamples were incubated with PBS-T based solutions of primary antibodies (Table 2). Primary antibody incubation was performed at room temperature for 2 days. Primary antibodies were washed off with PBS-T (48 h at room temperature with liquid exchange every 24 h). Secondary antibodies listed in Table 3 were applied at room temperature for 2 days. Tissue was then washed with PBS-T (48 h at room temperature with liquid exchange every 24 h) to remove excess secondary antibody.

**Table 2:** Primary antibodies used for immunohistochemical staining of cleared post mortem human brain tissue.

Cell Types/Compartments	Detected Protein	Antibodies	Dilution	Source
**Myelin and fibers**				
Myelinated fibers	Myelin basic protein (MBP)	Rat anti-MBP	1:400	Abcam
Microtubules	ß-III-Tubulin	Rabbit anti-ß-III-Tubulin	1:1 000	Sigma
**Cellular markers**				
Neurons	Human neuronal Protein C/D (HuC/D)	Mouse anti-HuC/D	1:400	Invitrogen
Fast spiking interneurons	Parvalbumin	Rabbit anti-PV25	1:500	Swant
**Glia markers**				
Microglia	Ionized calcium binding adaptor1 (Iba-1)	Rabbit anti-Iba1	1:500	Wako
Astroglia	Glial fibrillary acidic protein (GFAP)	Rabbit anti-GFAP	1:1 500	Dako
Astroglia	Glial fibrillary acidic protein	Mouse anti-GFAP	1:1 000	Sigma
Astroglia	Glial fibrillary acidic protein	Guinea-Pig anti-GFAP	1:500	Synaptic Systems
**Vessels**				
Vessel walls	α-D- and N-acetyl-Galactosamine residues	helix aspersa agglutinin, biotinylated	1:100	Sigma

**Table 3:** Secondary fluorescence labeled antibodies used for immunohistochemical staining of cleared post mortem human brain tissue.

Secondary antibodies	Dilution	Source
Donkey anti-mouse Cy2	1:150	Dianova
Donkey anti-mouse Cy3	1:200	Dianova
Donkey anti-rat Cy3	1:200	Dianova
Streptavidin Cy3	1:200	Dianova
Donkey anti-rabbit Cy5	1:150	Dianova
Donkey anti-guinea-pig Cy5	1:150	Dianova
Donkey anti-mouse Alexa 594	1:150	Dianova
Donkey anti-rat Alexa 647	1:150	Dianova

Additionally, three larger subsamples (1I, 1H and 2 A) were resected from tissue blocks and stained with HuC/D and GFAP (1I), with MBP (1H) and HuC/D (2 A) and used to study antibody penetration, cyto- and myeloarchitectonics using 3D optical imaging. For the staining of these larger samples the same procedure was used, but incubation time was prolonged (14 days for primary and 7 days for secondary antibody incubations). The prolonged incubation and rinsing times improved the results over previously published approaches.

For optical clearing and refractive index matching all samples were transferred into 47.5% 2,2′-thiodiethanol (TDE) (Sigma-Aldrich Co, LLC) PBS solution and finally mounted in cell culture dishes for 2D and 3D microscopy.

### MRI acquisition

High-resolution quantitative maps of longitudinal and effective transverse relaxation rates (*R1* and R2* respectively) were obtained for the both blocks of Case 1 at three stages of the clearing process (prior to clearing, after 2 weeks of clearing, after 15 weeks of clearing). Additionally, subsample 1H was imaged prior and after optical index matching step, but prior to staining. Scanning was performed on a whole body 7 T MRI scanner (Magnetom, Siemens, Erlangen, Germany) using a 32-channel receive coil and 1-channel transmit radio-frequency (RF) head coil. *R1* maps were acquired with the MP2RAGE sequence (Marques et al., 2010) with isotropic resolution of 0.36 mm × 0.36 mm x 0.36 mm, matrix size of 126 × 192 x 120, 3D acquisition, inversion times *TI*_1_/*TI*_2_ = 200/900 ms, repetition time *TR* = 3 s, echo time *TE* = 2.10 ms, flip angles *FA* = *α*1/*α*2 = 8°/8°, partial Fourier factor *PF* = 6/8 and pixel bandwidth *BW* = 338 Hz/Px. R2* maps were obtained from multi-echo FLASH images (3D acquisition, matrix 126 × 192 x 120, 6 echoes, *TE*_1-6_ = 4, 13.2, 22.4, 31.6, 40.8, 50 ms *TR* = 100 ms, *FA* = 29°, *BW* = 190 Hz/Px, *PF* = 6/8) with 0.3 mm isotropic resolution. R2* maps were obtained by linear log signal fit of the multi-echo images.

Width, length, thickness and volume of tissue blocks at all clearing stages were measured using MP2RAGE images.

### 3D microscopy

Two microscopy systems were used for 2D and 3D optical imaging of cleared tissue: a confocal laser-scanning microscope and a light-sheet microscope.

The Zeiss confocal laser-scanning microscope (LSM 510 Meta, Zeiss, Jena, Germany) was equipped either with a 10× CLARITY-objectives (Olympus XLPLN10XSVMP, numerical aperture (NA) 0.6, working distance (WD) 8 mm and Zeiss Clr Plan-Apochromat, NA 0.5, WD 3.7 mm) or with a 25× multi-immersion objective (Zeiss LCI Plan Neofluar, NA 0.8, WD 0.21 mm). The fluorescence was excited with an argon laser (488 nm, Cy2) and two HeNe lasers (543 nm, Cy3; 633 nm, Cy5). The emitted light was collected using three band-pass filters: 505–530 nm for Cy2, 565–615 nm for Cy3 and 650–710 nm for Cy5. The confocal laser-scanning microscope was used for 2D and 3D imaging of subsamples 1 B-I and 3 A.

The LaVision light-sheet fluorescence microscope (UltraMicroscope II, LaVision BioTec, Bielefeld, Germany) was equipped with a 10× CLARITY-objective (Olympus XLPLN10XSVMP), and operated with 630 nm excitation wavelength and band-pass 680 nm emission filter. The light-sheet microscope was used for 3D imaging of larger samples (1 A, 1H, 2 A). For the MBP-stained subsample 1H (sample size 20 mm × 20 mm x 8 mm) an image stack covering a 1.2 mm × 1.2 mm x 1.2 mm volume was acquired on a Region-Of-Interest situated about 1.2 mm from the sample edge and 1 mm deep from the sample surface (Fig. 6). The resulting TIFF image stack (16 bit, 2 560 × 2 160 pixels, 590 slices, 0.5 μm lateral resolution, 2 μm z-steps between slices) was split into regular cubes (0.6 mm × 0.6 mm × 0.6 mm) for further processing. This size corresponds to a typical size of MR voxel used in high field imaging *in vivo*. For the HuC/D-stained subsample 1 A (3 mm × 3 mm x 5 mm) an image stack covering a 2.6 mm × 2.2 mm x 2.6 mm volume situated 0.5 mm from the sample edge the was acquired (630 nm excitation wavelength, band-pass 680 nm emission filter, 5 ms exposure time, 16 bit, 5 120 × 4 320 pixels, 2 601 slices, 0.51 μm lateral resolution, 1 μm step size). The stack was split into four substacks (1.3 mm × 1.1 mm × 2.6 mm) for image processing. For the HuC/D-stained sample 2 A (5 mm × 5 mm x 5 mm) an image stack covering 2.2 mm × 2.6 mm x 5.1 mm volume and situated approximately in the middle of the sample was acquired (630 nm excitation wavelength, band-pass 680 nm emission filter, 5 ms exposure time, 16 bit, 2160 × 2 560 pixels, 510 slices, 1.02 μm lateral resolution, 10 μm step size).

### Microscopy image analysis for cytoarchitectonics

For the study of cyto-architectonics, 3D image substacks (1.3 mm × 1.1 mm x 2.6 mm) from the middle cortical depth of the HuC/D-stained sample (1 A) recorded with the light-sheet microscope were analyzed. Signal-To-Background ratio was determined for the images recorded at different image depth as a ratio of mean image intensity averaged over manually defined ROI's containing either cells or background.

In the first step machine learning approach was used to identify cell bodies from the background, artifacts and structures of no interest. We trained Random Forest classifier using the WEKA Trainable Segmentation (Arganda-Carreras et al., 2017) implemented in FIJI (see Table S1 for selected features) on the HuC/D-stained image stack to differentiate voxels belonging to cells or background. Two experts (NS and FG) labeled regions of foreground and background using a few slices from the top of the entire stack (taken from the first 10% of the images). We subsequently binarized the resulting 3D probability maps (using the Hysteresis threshold option in Ilastik setting core = 0.8 and final = 0.6 with all other options left at default values). Connected components were computed from the binary volumes and used in a second step for classification into pyramidal and non-pyramidal cells.

In the second step, the object classifier was trained in Ilastik (Sommer et al., 2011) using the standard object feature set and Random Forest classifier (Table S2) and manual expert classification on subset of cells.

Classification and validation were performed in the following way. To validate the first step one expert (FG) manually segmented 10 images (equidistantly distributed across imaging depth) to cells and background. These manual segmentations were compared with the results of the object classifier. Numbers of correctly identified cells were determined for all image depth. To validate the second step two experts (NS and SG [neuroanatomist with 26 years’ experience in histological analysis]) manually classified two randomly chosen subsets of neurons (independently chosen for each expert) into pyramidal and non-pyramidal cells based on shape, size and the presence of apical dendrite. The classified subset from NS was used to train the random Forest classifier. The classifier efficiency was estimated based on the comparison of automated classification with the manual classification of the second expert classifier (SG).

The final 3D maps were then computed using the centroids of all detected and labeled components.

### Microscopy image analysis for intracortical fiber tracking and fiber orientation distribution analysis

For the study of myelo-architectonics, 3D image stack (1.2 mm × 1.2 mm x 1.2 mm volume) obtained from middle cortical depth of an MBP-stained subsample (1H) recorded with light-sheet microscope was analyzed.

In the first step, we used a machine learning approach to identify fiber structures and distinguish them from the background, artifacts and structures of no interest in the 3D microscopy data. A machine learning classifier has been trained on a sample of 10 sub-regions (400 × 400 pixel) randomly selected from the original stack using the WEKA Trainable Segmentation (Arganda-Carreras et al., 2017) implemented in FIJI (Schindelin et al., 2012). We used a subset of the available image features (Table S1) and a standard Random Forest classifier from the WEKA plug-in for training. Two experts (NS and FG) labeled regions of foreground and background using a few slices from the top of the entire stack (taken from the first 10% of the images). The trained classifier (97.63% correct classification tested with 10 fold cross-validation) was applied to the entire stack and probability maps for the class corresponding to fiber structures were extracted for analysis. Multi-scale vessel enhancement filtering (Frangi et al., 1998) was used to suppress all remaining non-tubular structures in the probability maps (using 5 scales from 0.5 μm to 2.5 μm).

In the second step, the in-plane orientation of fiber structures was obtained from the probability maps using the local edge orientation computed by the Directionality module in FIJI or alternatively using the estimation of local structure tensor algorithm implemented in OrientationJ (Püspöki et al., 2016). Orientation histograms were computed separately for each image plane along the stack. The results were visualized using a custom script for Mathematica 11 (Wolfram Research, Champaign, IL).

Due to the anisotropic resolution of optical imaging, the analysis of fiber orientation was performed in only in 2D.

## Results

### Optimized CLARITY on large samples preserved tissue fine structure and protein content

**Fig. 1:**
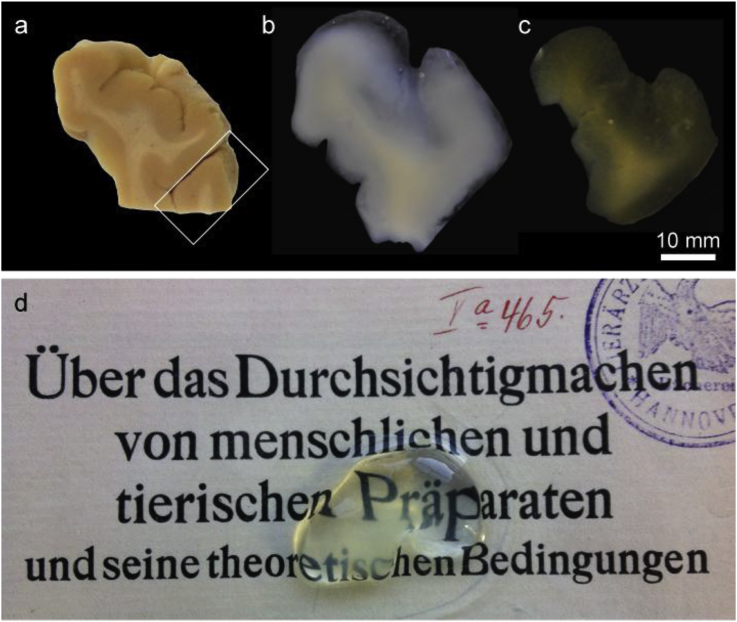
Post mortem human brain tissue block (Case 1) at different stages of clearing process. 8 mm thick sample from temporal lobe prior to clearing (a), after 7 months of CLARITY (b), after 10 months of CLARITY and optical index matching in TDE (c). (d) Part of the block (indicated with white frame in (a)) after 14 months of CLARITY and refractive index matching on the title page of the Werner Spalteholz’ publication on the first tissue clearing method (Spalteholz, 1914).

After three to fourteen months of active and passive clearing all brain tissue blocks became visually transparent (Fig. 1). Compared to white matter, grey matter was cleared faster and reached higher overall transparency.

**Fig. 2:**
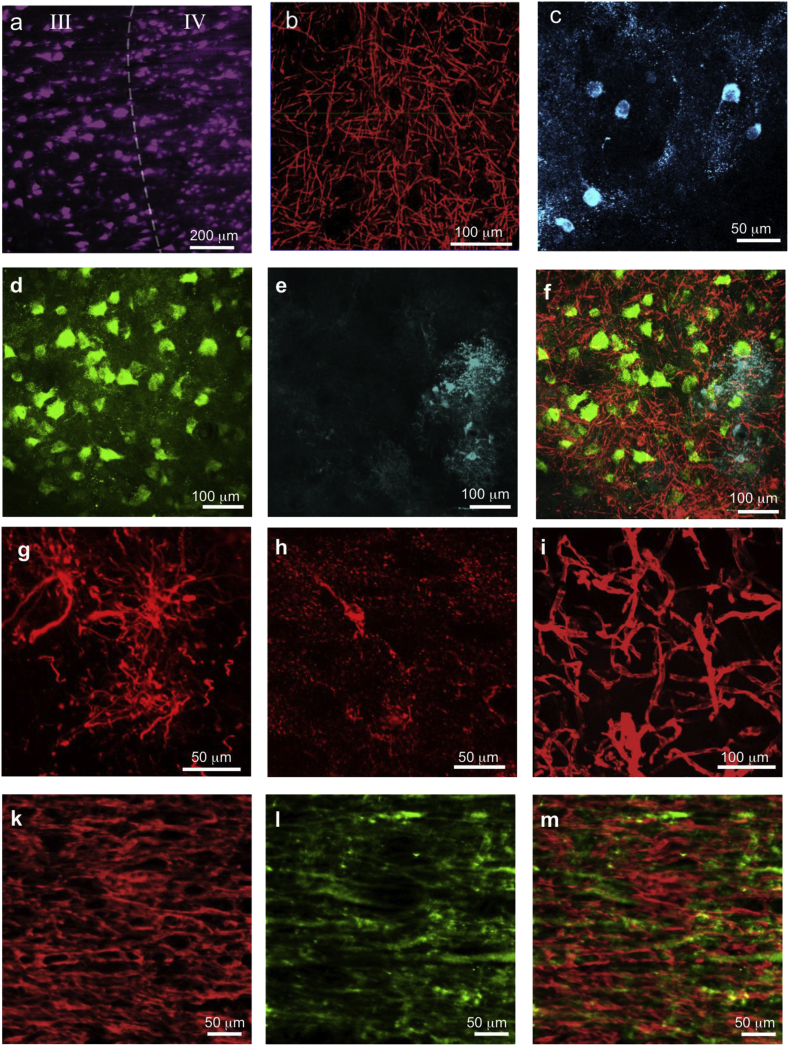
Immunohistochemical staining of human post mortem brain tissue after clearing. High contrast of all stains reveals preserved protein composition of the sample despite lipid loss upon intensive clearing. (a) Cortex subsample (1 A) with 3 mm × 3 mm x 5 mm size stained with neuron marker HuC/D. Cortical layers III and IV can be identified based on neuronal morphology. See Video S1 for 3D version of entire imaging volume; (b) Myelinated fibers in the cortex subsample (1 B) stained for protein component MBP (myelin basic protein, 3 mm × 3 mm x 3 mm sample size). See Video S2 for 3D version of entire imaging volume; (c) Subsample (1C) stained for parvalbumin, a calcium binding protein for sub-classification of neurons, especially to identify fast spiking interneurons; (d–f) Cortex subsample (1D) triple stained for neurons ((d), HuC/D, green), astrocytes ((e), GFAP, cyan) and myelinated fibers (MBP, red); (f) overlay; (g) Cortex subsample (1E) stained for astrocytes (GFAP, red); (h) Cortex subsample (1F) stained for microglia (Iba-1, red); (i) Vessels in the cortex subsample (1G) stained with helix aspersa agglutinin; (k,l,m) Double labeling of corpus callosum subsample (3 A) stained for (k) MBP and (l) β-III-tubulin; (m) overlay of both stains. Imaging of corpus callosum (k, l, m) was performed in PBS (all other samples were TDE embedded), thus tissue is 1.25 times expanded as compared to its original size. Image (a) is acquired with light-sheet microscope, images (b–m) are acquired using laser scanning microscope.

The immunohistochemical stains demonstrated that, despite lipid removal, most tissue proteins were well preserved, allowing a visualization of multiple cellular and fiber compartments (Fig. 2).

Neuronal cell bodies were stained using HuC/D allowing visualization and identification of neurons in temporal lobe grey matter (Fig. 2a, Video S1, Video S5) throughout the sample thickness of up to 2.6 mm for subsample 1 A (Video S1) and up to 5 mm for subsample 2 A (Video S5). Myelinated fibers in grey matter were well identified on the MBP stains (Fig. 2b, f, Videos S2, Video S3) up to the 1.2 mm depth within tissue. The successful staining for parvalbumin, a calcium binding protein, enabled us to identify fast spiking interneurons (Fig. 2c).

Supplementary video related to this article can be found at https://doi.org/10.1016/j.neuroimage.2017.11.060.

The following are the supplementary data related to this article:

Video S1 Video S1

Video S2 Video S2

Video S3 Video S3

Video.S5 Video.S5

**Fig. 3:**
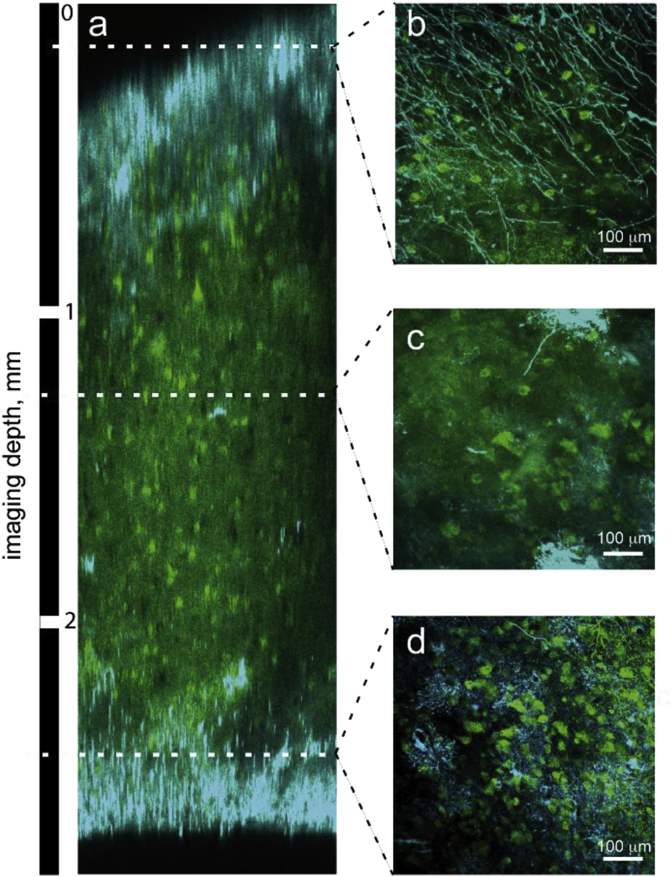
Immunohistochemical staining efficiency of 3 mm thick subsample (1I). (a) Reconstruction of a fast z-line scan over the cortical profile with selective stacks (z = 100 μm) of upper (b), middle (c) and deeper (d) layers of a 3 mm thick cortex tissue block. Neurons (HuC/D, green) and astrocytes (GFAP, cyan) are visible throughout the block with slight variations in staining intensity. Upper stack (b) shows fibers of cortical layer I.

Astrocytes were clearly visible in GFAP stains (Fig. 2e, f, g) up to the depth of 3 mm (Fig. 3).

Microglia were well visible in the Iba-1 stain (Fig. 2h). Intracortical vessels could be delineated using helix aspersa agglutinin lectin stain (Fig. 2i).

In dense white matter identification and tracking of single fibers was feasible with the β-III-tubulin fiber stains (Fig. 2l and m). In MBP stained subsamples 3 A the identification of single fibers in white matter was hampered due to the dense appearance of white matter and degradation of myelin sheaths upon active clearing or prolonged post mortem delay (Fig. 2k,m).

The efficiency of immunohistochemical stains for a 3 mm-thick subsample 1I is shown in Fig. 3. A fast z-line scan profile of the sample double stained with HuC/D for neurons and GFAP for astrocytes shows homogenous staining throughout the complete sample thickness of 3 mm. Identification of neuron bodies was possible on the surface, in the middle and in the depth of the sample (Fig. S2). Also astrocytes could be identified at the cortical surface (Fig. 3b), in the middle of the cortex (Fig. 3c) and at the border to white matter (Fig. 3d). GFAP staining revealed a dense layer of glia limitans and characteristic interlaminar glia in the superficial cortical layer, high density of astroglia in the white matter as well as astroglia cells in the grey matter (Fig. 3b,c,d).

Thus, we can conclude that efficient immunohistochemical staining was achieved through the entire cortex up to a depth of at least 5 mm for HuC/D stain and of at least 3 mm for GFAP stain and of at least 1.2 mm for MBP stain.

### Tissue expands and MR contrast vanishes upon clearing

**Fig. 4:**
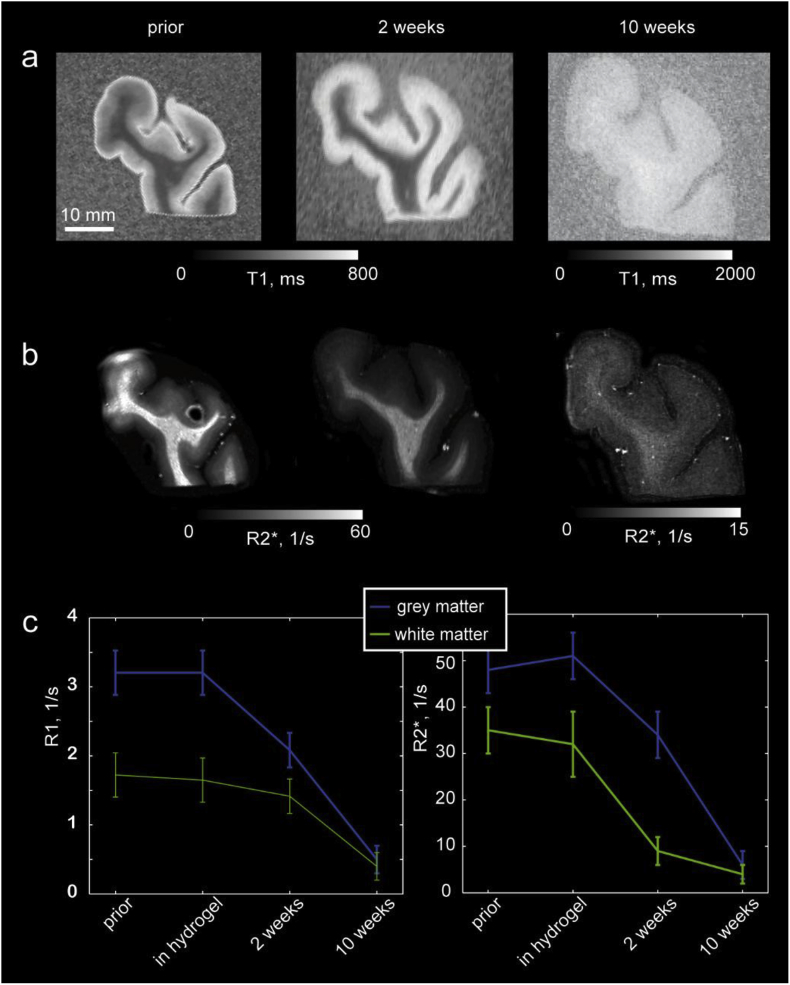
MRI contrast changes upon tissue clearing. Quantitative T1 (a) and R2* maps (b) of a tissue block (Case 1) at three stages of the clearing process (left: prior to clearing, middle: after 2 weeks of clearing, right: after 10 weeks of clearing) for the brain tissue sample from temporal lobe. Note the tissue expansion at later clearing stages. (c) Averaged R1 (=1/T1) and R2* values for grey and white matter at different stages of tissue clearing. MR contrast between grey and white matter disappeared after lipid removal in line with the strong contribution of myelin to T1 and T2* contrasts.

Prior to clearing formalin-fixed brain tissue showed exquisite contrast between white and grey matter in both *R1* (=1/T1) and R2* (=1/T2*) quantitative maps (Fig. 4a b, left column). Obtained values for *R1* and R2* (grey matter: *R1* = 2.1 ± 0.2 s^−1^, R2* = 19 ± 6 s^−1^; white matter: *R1* = 3.3 ± 0.2 s^−1^, R2* = 48 ± 4 s^−1^) were in good agreement with literature values (Stüber et al., 2014). Hydrogel embedding did not change *R1* and R2* significantly (Fig. 4c). However, both relaxation rates R1 and R2* were strongly decreased by clearing and tissue expansion in hydrogel (Fig. 4a and b middle and right columns, Fig. 4c). The contrast in *R1* and R2* vanished almost completely in the final clearing stage. Interestingly, the contrast between grey and white matter partly re-appeared after embedding in TDE for optical index matching (Fig. S1).

Extensive tissue expansion (up to 145% increase in size and 300% increase in volume) was observed after hydrogel hybridization and during SDS clearing stage (Fig. 1, Fig. 4), but the tissue shrunk back to its original size (92% ± 20% volume changes) after incubation in 47.5% TDE for optical index matching (Fig. 1c, S1).

### Cytoarchitectonics in 3D

**Fig. 5:**
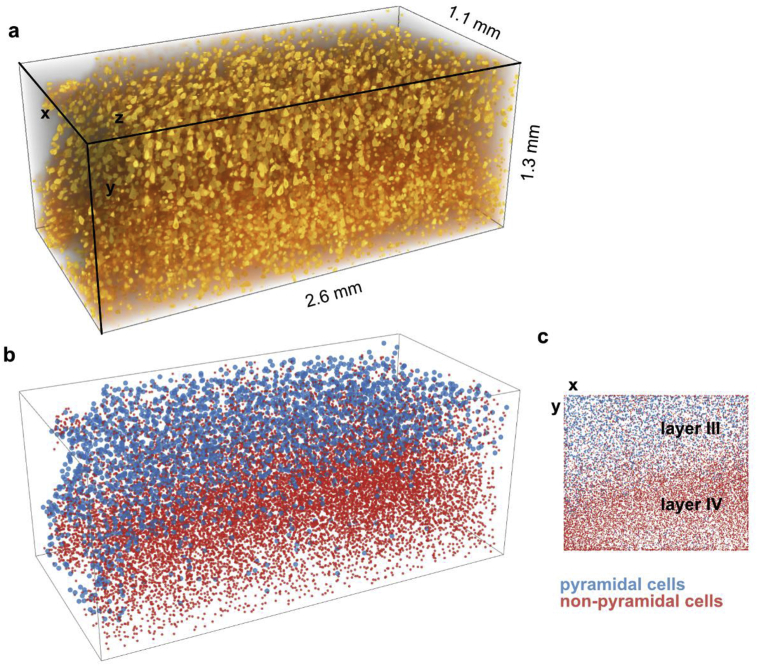
Mapping of cortical cyto-architectonics in 3D within the volume of typical MRI voxel. (a) Reconstruction of a 3D image of cortex subsample (1 A) with neuronal cell bodies stained for HuC/D. Y-axis corresponds to the direction from white matter to pial surface and z-axis corresponds to the imaging depth direction. (b) Results of classification of neuronal cell body morphometry to pyramidal (blue) and non-pyramidal (red) neurons. Cortical layers III and IV are clearly identified throughout the entire imaged volume of 2.6 mm indicating a consistent imaging quality throughout the sample. (c) Projection of classified volume over the z-direction. Layers III and IV can be clearly recognized.

Comprehensive 3D cytoarchitectonics were extracted from the subsample 1 A stained with HuC/D for neurons and scanned with the LaVision light-sheet microscope (Fig. 2a, Video S1, Fig. S2). Images of high quality were obtained for a depth into the tissue of up to 2.6 mm (Video S1, Fig. S2) with a signal-to-background-ratio varying from 4.4 close to the surface to 3.6 at the image depth of 2.2 mm (Fig. S2). The identification of pyramidal and non-pyramidal neurons was possible across the entire inverstigated subvolume (size of 1.3 mm × 1.2 mm x 2.6 mm) (Fig. 2, Fig. 5, Fig. S2, Video S1). Two clear cortical layers with different densities of pyramidal and non-pyramidal cells corresponding to cortical layer III and IV were visible throughout the entire image depth (Fig. 5b and c). A quantitative cytoarchitectonic analysis was performed. Density maps of pyramidal and non-pyramidal cells were generated. Based on the densities of pyramidal and non-pyramidal cells two cortical layers (III and IV) were identified throughout the entire image depth (Fig. 5b and c). Layer III was assigned as areas with higher density of non-pyramidal, layer IV with higher density of pyramidal neurons.

A classifier trained to separate pyramidal and non-pyramidal type neurons achieved 95.4% classification accuracy with no systematic variation across the entire depth (see Table S3 and S4 in Supplementary), corroborating the high and consistent image quality. Based on the layer segmentation of the image volume 48.8% of the volume was assigned to the layer III and 51.2% to layer IV.

### Fiber orientation distributions

**Fig. 6:**
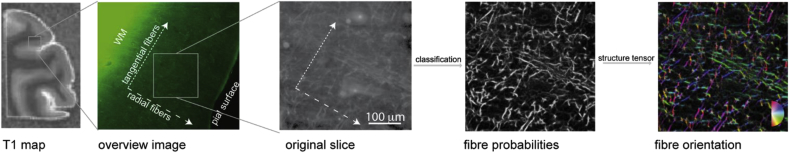
Overview of image analysis steps for intracortical fiber characterization. A single original slice (subsample 1H) from the image volume at 1.2 mm depth (middle picture, scale bar 100 μm) is used to generate a probability map computed by a random forest classifier trained on fibre structures. Orientation of fiber (far right picture) structures is computed by analyzing the local structure tensor at each voxel position.

**Fig. 7:**
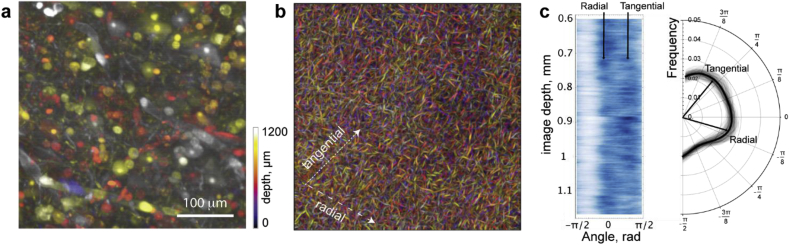
Classification algorithm enhances linear structures and allows studying fiber orientation distribution within a volume similar to an MRI voxel volume. (a) Original data (subsample 1H) projected by a color-coded maximum intensity projection (color represents imaging depth, see scale). (b) The processed stack (after classification and vesselness filtering) shown in the same projection as in (a). Orientation of fiber structures in the image volume: (c) Fiber orientation distribution. Heat map shows the directionality (in-plane) distribution at each slice along the depth of a 600 × 600 × 600 μm^3^ volume at an imaging depth of 600 μm. Slices are running at the constant cortical depth. Circular histogram showing the distribution of fibre directionality averaged over the entire 600 μm range. Major fibre directions (corresponding to radial and tangential fiber orientations with respect to cortical) are indicated by arrows in (b).

The fiber density and fiber orientation distribution were analyzed in the 3D image stack of the cleared cortex from the temporal lobe subsample 1H stained for MBP. The size of the stack obtained with the LaVision light-sheet microscope was 1.2 mm × 1.2 mm x 1.2 mm, which is comparable to or even larger than the voxel sizes currently used in advanced DWI *in vivo* (Heidemann et al., 2012, Setsompop et al., 2017, Setsompop et al., 2013, Sotiropoulos et al., 2013, Vu et al., 2015). The investigated sub-volume covering cortical layers IV-V showed high intracortical fiber density (Fig. 6). Fibers oriented radially and tangentially relative to the cortical surface were clearly visible on the images throughout the imaged sub-volume (Fig. 6, Video S4). Image artifacts resulting from auto-fluorescence of cell and blood vessels as well as stripes resulting from the scattering and absorption of the light-sheet in the tissue were detectable in the raw images (Figs. 6 and 7, Video S4 left panel). The classification algorithm combined with the vesselness filter was able to remove most of the image artifacts (Figs. 6 and 7a and b) and provided estimated fiber probability maps in 3D. Fiber orientation distributions obtained from the imaged volume are shown in Fig. 7. They were restricted to 2D due to the anisotropic image resolution (0.5 μm × 0.5 μm in plane and 4 μm through plane). The fiber orientation distribution was obtained by averaging of information extracted from 2D images across the imaged volume.

Supplementary video related to this article can be found at https://doi.org/10.1016/j.neuroimage.2017.11.060.

The following is the supplementary data related to this article:

Video S4 Video S4

## Discussion

We propose an advanced histological approach, which can be used to inform and validate methods for *in vivo* histology using MRI. The approach combines quantitative MRI, tissue clearing, fluorescent staining, 3D optical imaging and image analysis and can be applied to aged post mortem human brain tissue. Using an optimized CLARITY procedure we demonstrated the clearing of samples with the thickness of up to 8 mm and an efficient immunohistochemical staining of post mortem human brain tissue samples with a thickness of up to 5 mm (Fig. 1, Fig. 3 and Video S5). We have shown that the procedure exquisitely preserves the tissue's microstructure and protein content (Fig. 2), thus enabling the comprehensive analysis of microstructure, cyto- (Fig. 5) and myeloarchitectonics (Fig. 7) of macroscopic human cortex samples in 3D. We demonstrated that the achieved tissue transparency facilitates single fiber identification and structure tensor analysis across a volume corresponding to the voxel size of state-of-the-art DWI acquisitions or even larger volumes. This approach promises to help integrating MRI-based and optical microscopy in 3D, and enable the further development of *in vivo* histology using MRI.

Our results have several important implications. Firstly, we demonstrated improved performance of the adapted CLARITY method on aged human post mortem material compared to previous reports (Table 4). Compared to these reports we significantly increased the thickness of the cleared tissue (8 mm vs. 3 mm reported before) and improved the depth of the immunohistochemical staining and optical imaging (5 mm vs. 1 mm). Additionally, we demonstrated for the first time the feasibility of immunohistochemical stains for HuC/D, β-III-tubulin, parvalbumin and helix aspersa agglutinin in challenging aged post mortem human brain tissue. The larger field of view of the 3D microscopy may make it possible to study mesoscopic structures of up to several millimeters extent, such as the entire cortical profile, stripes in the visual cortex as well as intracortical fibers along their entire extent.

**Table 4:** Summary of published literature on CLARITY applied to post mortem human brain tissue.

Reference	Age, pathology, fixation	Thickness/brain area/clearing procedure/image analysis	Staining
Chung et al. (2013)	7 y/male/autism	500 μm frontal cortex, Brodmann area 10 active clearing	MBP, parvalbumin neurofilament, GFAP tyrosine hydroxylase
Liu et al. (2016)	Parkinson patient	3 000 μm (imaging up to 771 μm) cerebellum passive clearing	neurofilament Iba-1, α-synuclein tyrosine hydroxylase
Ando et al. (2014)	5 AD patients 2 controls	500 μm frontal cortex passive clearing	anti-tau B19 anti-Aβ 4G8 neurofilament
Phillips et al. (2016)	2 controls 5 mitochondrial disease patients	250 μm cerebellum passive clearing	MBP neurofilament mitochondria COXI Glut-1 α-smooth muscle actin
Costantini et al. (2015)	10 y/child hemimegalencephaly	2000 μm (imaging up to 1 000 μm) passive clearing intracortical fiber tracking for 1 mm	parvalbumin nuclei (DAPI) GFAP
(Leuze et al., 2016)	no information available	500 μm thalamus, passive clearing structural tensor analysis	neurofilament
Current study	54-70 y/3 controls no neurological diseases	8 000 μm transparency, temporal lobe, corpus callosum passive and mild active clearing (imaging 2.5 × 2.5 × 4 mm^3^) fiber orientation distribution cell classification	MBP, HuC/D, GFAP, ß-III-tubulin, Iba-1, parvalbumin, helix aspersa agglutinin

Secondly, we demonstrated that the proposed method provides information, which can be used to inform and validate MRI histology in humans. Importantly, the achieved imaging volumes are larger than the typical voxel sizes in state-of-the-art MRI. Therefore the 3D microscopy images can be used for biophysical modeling of the MRI contrast (from the underlying microstructural feature), which arises as an aggregate measure from the entire voxel.

The fiber orientation distributions estimated from the 3D histology data (Fig. 7) can be directly compared to model parameters extracted from DWI and MR-based myelin mapping methods (Leuze et al., 2014, Weiskopf et al., 2015). Several histological methods were used for validation of DWI in the past (Budde and Annese, 2013, Magnain et al., 2014, Magnain et al., 2015, Schilling et al., 2016, Seehaus et al., 2015, Wang et al., 2014). Generally, these studies were based on 2D microscopy and processing of consecutive slices. Two main drawbacks of these methods are the missing information in the third dimension and necessity to register multiple slices. The latter is challenging, since it requires sequential histological processing and elastic co-registration of multiple 2D slices unless a block face imaging approach is used (Magnain et al., 2014, Magnain et al., 2015). Using CLARITY thick 3D stacks can be imaged without challenging co-registration of consecutive slices. Co-registration between MR images of intact tissue before and optical images after clearing is a remaining challenge, taking into account tissue distortions upon clearing. However 3D co-registration algorithms may be used for this purpose, which are more robust than 2D to 3D registration algorithms.

### Considerations

Several challenges need to be addressed in order to further improve the image quality, increase sample size and imaging volume. Previously, tissue opacity and low antibody penetration were reported to be the main limiting factors for imaging of thicker sections (Liu et al., 2016). We demonstrate that larger samples can be cleared and stained when combining mild active and passive clearing, prolonged clearing and incubation times. Remaining light scattering in the tissue and inhomogeneity of the refractive index induced some distortions in the optical images (Video S2) and potentially broadened the point-spread-function in imaging direction at larger imaging depths. The requirements on sufficient sample transparency and optical index homogeneity depend strongly on the structures of interest, sample thickness, immunohistochemical stain and image processing algorithms for a specific application. Structures with the size of several micrometers such as vessels and neuronal cell bodies are more easily detectable even in a greater imaging depth. However, densely packed myelinated fibers with sizes of ≥1 μm pose significantly higher requirements on sample transparency and resolution of the optical imaging system. Also, different 3D optical imaging technologies place different requirements on the sample optical properties. Thus, no simple universal requirements on tissue transparency can be defined. Rather the successful performance of image processing algorithms for the particular application may be best used as a performance indicator of the workflow. Here, we demonstrated robust classification of neurons at the depth of 2.6 mm and of myelinated fibers at 1.2 mm depth.

Both confocal laser scanning microscopes and light-sheet microscopes provide 3D data with a nominal resolution that is much higher in plane (0.5 μm) as compared to through-plane (4 μm for light-sheet system and roughly 1–4 μm or thicker for laser the scanning setup without deconvolution (Power and Huisken, 2017)). The anisotropy of the point-spread-function constitutes severe challenges for the analysis of fiber orientation in 3D. The limited and anisotropic resolution can hinder the detection and precise localization of structures, particularly the densely packed intracortical fibers. In our experiments it affected the precise characterization of the 3D distribution of myelinated fibers, since the high anisotropy of the point-spread-function along the optical axis introduced a strong bias in the estimated directions. This is particularly prominent when the size of the fiber (0.5 μm) is much smaller than the point spread function in the imaging direction (at least 4 μm for the light-sheet microscope). Thus, we estimated the in-plane fiber orientation distributions only. This drawback is mainly due to the limitations of the 3D optical imaging system used in our study. This inherent limitation of imaging systems can only be overcome by using dedicated microscopy systems facilitating multi-view imaging, like the adopted version of dual inverted selective plane illumination microscopy (diSPIM) for large samples (20 mm × 20 mm x 8 mm) (Kumar et al., 2014, Wu et al., 2013) and respective multi-view de-convolution algorithms (Preibisch et al., 2014, Schmid and Huisken, 2015).

A precise co-registration of the microscopy and MRI data will be required in future studies targeting interfaces and transitions between small structures such as cortical layers. This might be challenging considering the tissue distortion upon clearing. Structural landmarks visible in MRI and in microscopy (e.g. highly myelinated layer IV, blood vessels, Fig, 2i) may facilitate the registration. Unfortunately, the MRI contrast between grey and white matter almost disappears after lipid removal, as also observed by Leuze et al. (2017). in the mouse brain. However, we also observed some recovery of grey-white matter contrast after immersion in TDE together with the tissue shrinking back to the original size. Both effects are expected to facilitate the registration. The contrast recovery indicates that the MR contrast in aged human brains is also driven by other factors potentially such as protein accumulations and varying water content.

The presented method provides important insights into the tissue microstructure, but does not provide quantitative information on absolute iron or myelin concentrations, which is required for modeling of several MR contrasts, such as T1, T2*, T2 and magnetization transfer contrasts. Iron is washed out by the clearing process (Leuze et al., 2017). Absolute myelin concentration and absolute fiber volume fraction are not accessible by immunohistochemical stains and 3D optical imaging with the presented resolution. The strength of the CLARITY method is that it provides information on tissue geometry and fiber orientation distributions. Thus the most promising application of the CLARITY method in the field of MRI-based microstructure imaging is the validation and biophysical modeling of DWI-based methods.

Currently, a powerful histological method for validation of DWI imaging in post mortem brain is PLI (Leuze et al., 2014, Mollink et al., 2017). CLARITY and PLI provide complementary information. The advantage of CLARITY and immunohistochemical staining is the possibility of comprehensive histological analyses and single fiber tracking. However, it requires handling and analysis of large datasets for comparison with DWI measurements. PLI operates at lower resolution and, similar to DWI, provides information on averaged tissue characteristics. This is beneficial when the two methods are compared but limits information about fine microstructural details.

Long clearing intervals are another drawback of the presented approach, which may limit its fast applicability, particularly when even thicker samples have to be cleared.

Several other clearing methods have been recently successfully applied to human brain tissue (Liebmann et al., 2016). In this work we used the CLARITY method for tissue clearing, since it provides the highest flexibility in terms of an overall spectrum of immunofluorescent labeling. However, other clearing techniques may outperform CLARITY depending on the particular applications and scientific objectives.

An important future challenge is to transfer the described method to even larger samples, ideally entire brains or hemispheres, with a planar extent of several centimeters. This step requires dedicated optical imaging systems that are able to image the entire sample with subcellular resolution in a reasonable time and are further able to cope with increased scattering and refraction deep within large tissue samples. Adaptive microscopy might be needed to overcome at least some of these limitations in the future (Ji, 2017, Royer et al., 2016, Scherf and Huisken, 2015). Furthermore, the amount of acquired data from large specimens poses severe problems to current analysis pipelines and data management systems, and might require the development of intelligent and adaptive analysis schemes.

The presented approach addresses the challenge of bridging the gap between mesoscopic resolution MRI with large imaging volumes and microscopic resolution histology with very small imaging volumes (or sections). It promises to enable the direct comparison between MRI-based and microscopy-based 3D histology on aged post mortem brain samples. Progress in this field would be an important step towards the further development and validation of quantitative MRI and hMRI. In addition, it has the potential to provide important anatomical reference data ranging from the microscopic to the macroscopic scale.
