# Supplementary material for: Developing 3D microscopy with CLARITY on human brain tissue: Towards a tool for informing and validating MRI-based histology
Source: Neuroimage. 2018 Nov 15;182:417–28. doi: 10.1016/j.neuroimage.2017.11.060 (PMC6189522; doi:10.1016/j.neuroimage.2017.11.060)
Supplement: Supplementary data [file mmc1.docx]

SUPPLEMENTARY MATERIAL

*
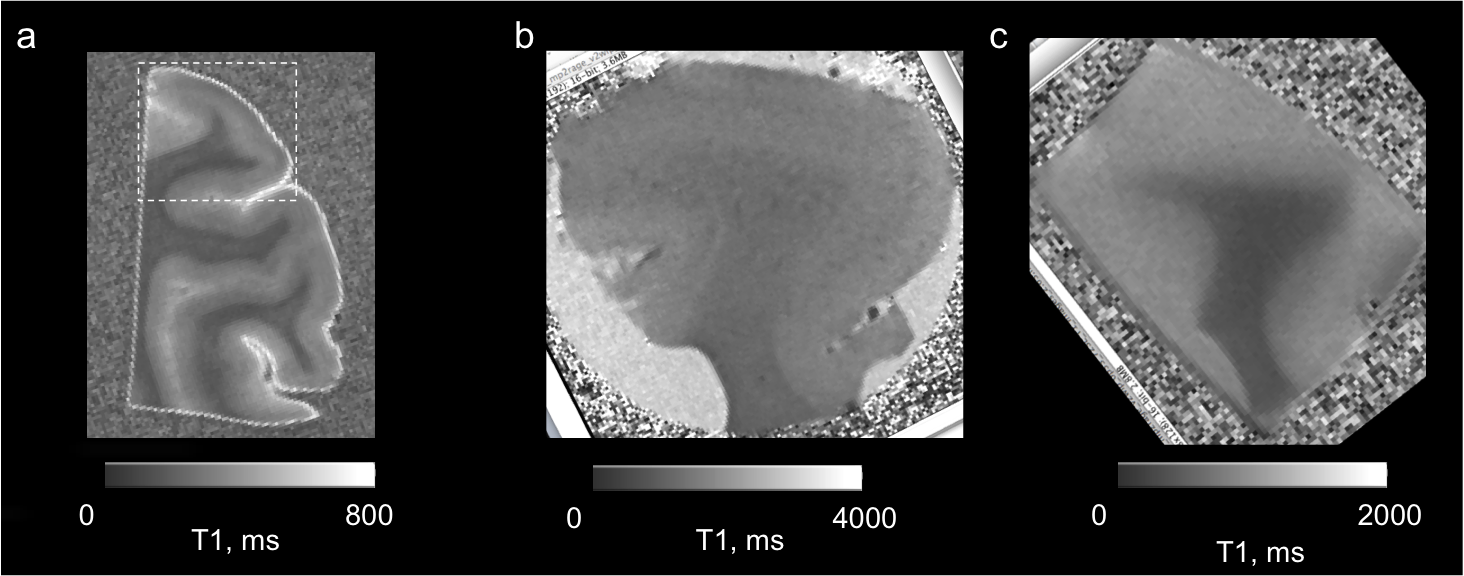
*

Figure S1. MRI contrast changes upon tissue clearing and optical index matching. Quantitative T1 maps of a tissue block (Case 1) (a) before clearing, (b) after 6 months of clearing (subsample 1H indicated in [a] with dotted box), (c) after TDE embedding (same subsample). MRI contrast between grey and white matter vanished after lipid removal but partly re-appeared after TDE embedding.


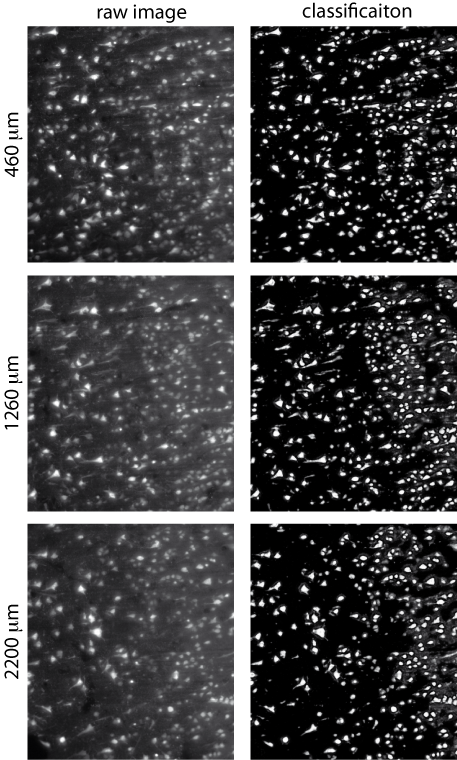


Figure S2. Image quality and efficiency of the cell-classifier at different imaging depths. (left) Images obtained from a HuC/D stained subsample 1A using the light sheet microscope at the depth of (from top to bottom) 460 μm, 1260 μm and 2200 μm. Signal-To-Background ratio was 4.4 (460 μm), 3.8 (1260 μm) and 3.6 (2200 μm) respectively. (right) Classification results.

| **classified compartment** | **image features** |
| --- | --- |
| Fibres | - Gaussian blur (5 scales from 1.0 to 16) - Difference of Gaussians - Hessian - Anisotropic Diffusion - Variance - Structure - Neighbors - Membrane Projections (thickness set to 5, patch size: 19) - Gabor |
| Cells | - Gaussian blur (5 scales from 1.0 to 16) - Difference of Gaussians - Hessian - Sobel - Membrane Projections (thickness:1, patch size: 19) |

Table S1: Trainable Weka Segmentation in FIJI. Local image features used for training fibre and cell classifiers.

| **feature class** | **features** |
| --- | --- |
| Intensity | - Histogram (+Neighborhood) - Kurtosis (+Neighborhood) - Maximum (+Neighborhood) - Mean (+Neighborhood) - Minimum (+Neighborhood) - Quantile - Skewness (+Neighborhood) - Total (+Neighborhood) - Variance (+Neighborhood) |
| Shape | - Size - Principal components - Radii |
| Location | - Bounding Box Maximum - Bounding Box Minimum - Center |

Table S2: Features for neurons classification using object classification in Ilastik.

| **Imaging depth (mm)** | **Percentage of cells from reference detected correctly** |
| --- | --- |
| 0,040 | 85,5 |
| 0,308 | 91,3 |
| 0,570 | 90,7 |
| 0,824 | 91,5 |
| 1,080 | 89,6 |
| 1,328 | 93,9 |
| 1,582 | 92,9 |
| 1,840 | 92,8 |
| 2,108 | 91,0 |
| 2,368 | 92,3 |

Table S3: Validation of cell detection step (used within Ilastik workflow).

| **Imaging depth (mm)** | **Number of Correct cell type prediction** | **Number of False cell type prediction** | **Percentage correct** |
| --- | --- | --- | --- |
| 0,400 | 17 | 0 | 100 |
| 0,800 | 13 | 3 | 81 |
| 1,200 | 15 | 0 | 100 |
| 1,600 | 13 | 0 | 100 |
| 2,000 | 14 | 0 | 100 |
| 2,400 | 11 | 1 | 91,6 |
| Total: | 83 | 4 | 95,4 |

Table S4: Validation of cell type prediction using Ilastik.


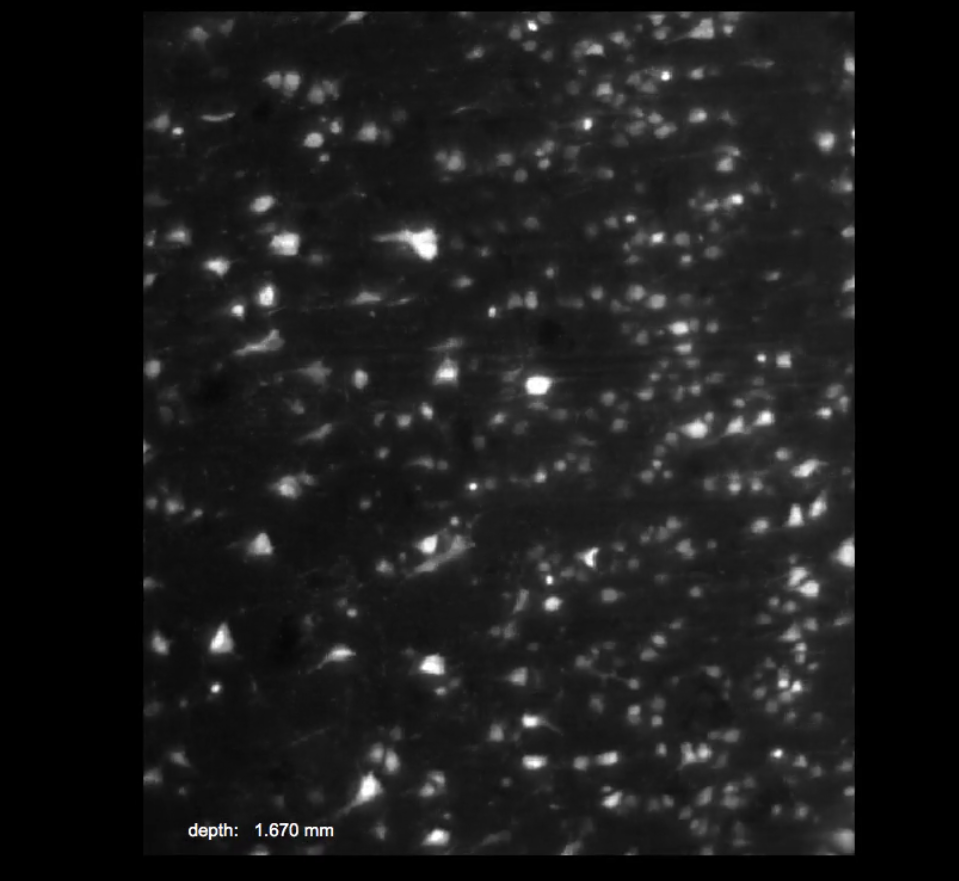


Video S1. A 1.2 mm x 1.2 mm x 2.5 mm stack of post mortem human cortex subsample (1A) stained for neurons (HuC/D) and recorded with the light sheet microscope. Pyramidal and granular cells are well delineated throughout the entire imaging volume. This volume was used for the classification of neurons shown in Fig. 5.


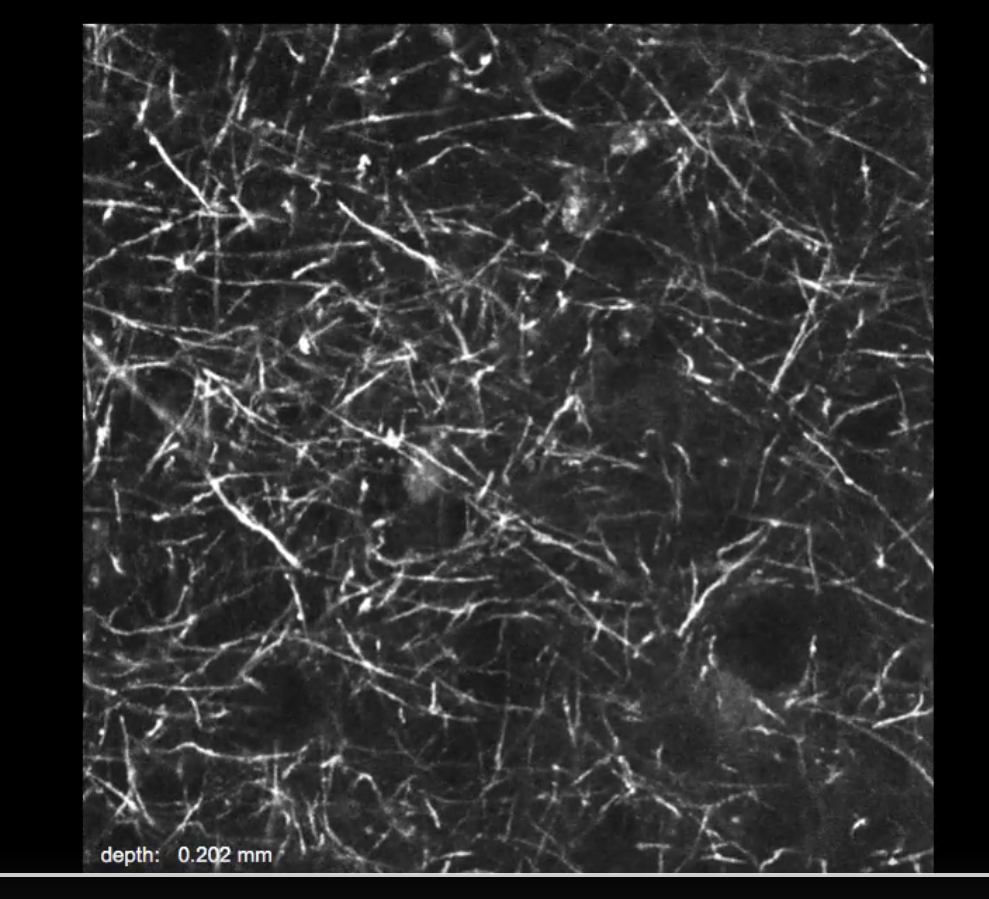


Video S2. A 230 μm x 230 μm x 200 μm stack of post mortem human cortex subsample (1B) with myelinated fibers stained (MBP) recorded with laser scanning microscope (Zeiss). Myelinated fibers are identified throughout the imaging volume.


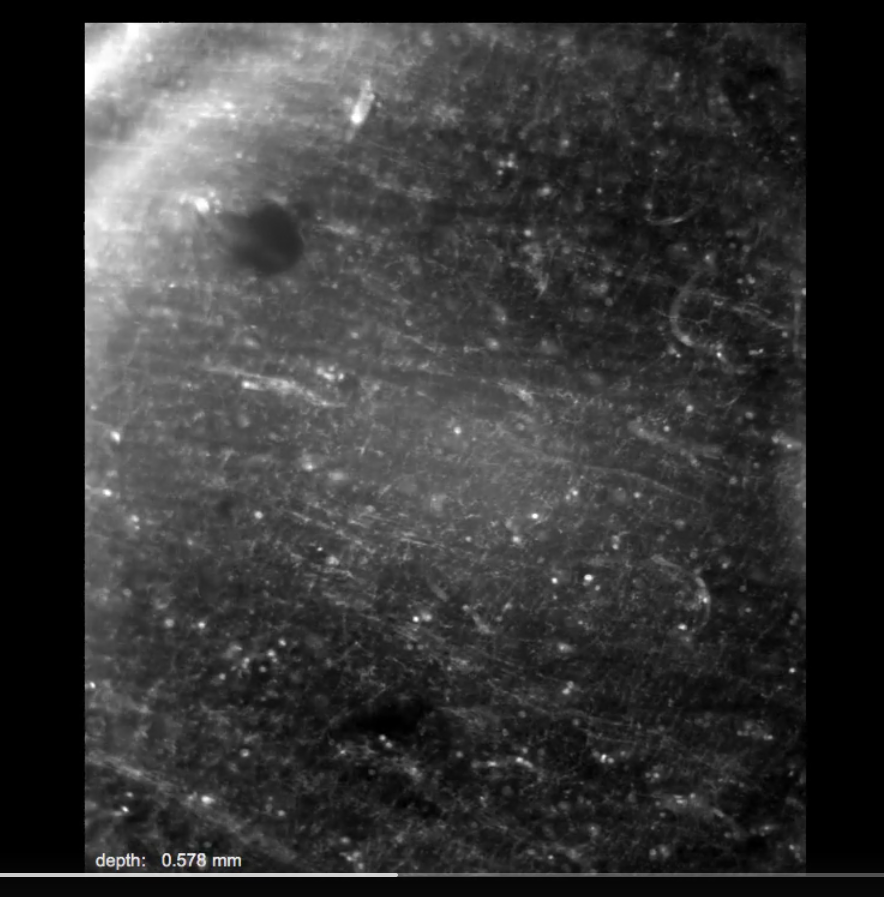


Video S3. A 1.2 mm x 1.2 mm x 1.3 mm stack of post mortem human cortex subsample (1H) stained for myelinated fibers (MBP) recorded with the light sheet microscope (LaVision). Myelinated radial and tangential fibers are seen throughout the imaging volume. Orientation of the sample and directions of radial and tangential fibers are shown in Fig 6.


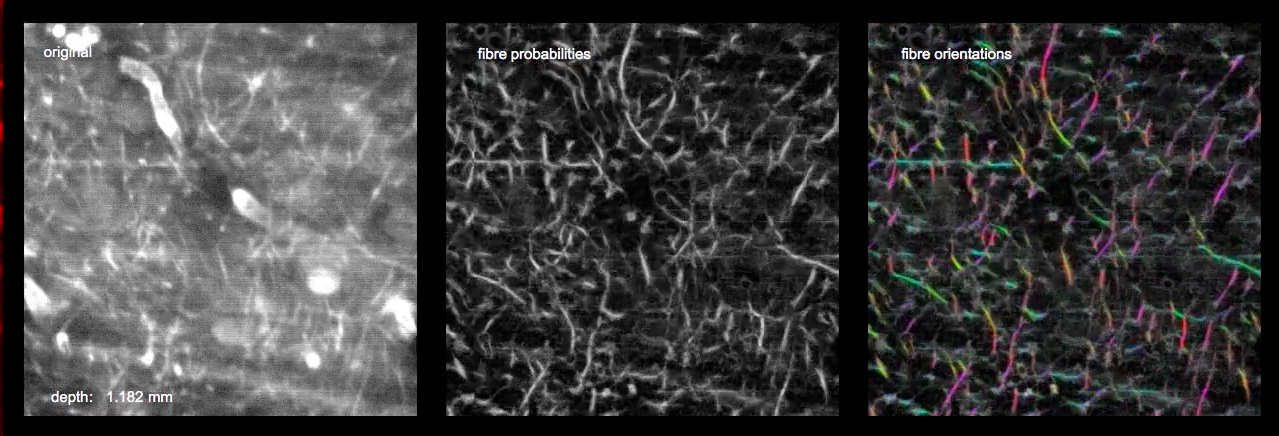


Video S4. A 400 μm x 400 μm x 1182 μm sub-volume of a stack segment (full stack presented in Video S3) stained for myelinated fibers (1H, MBP) (left) together with results of fiber classification (middle) and fiber orientation analyses (right).


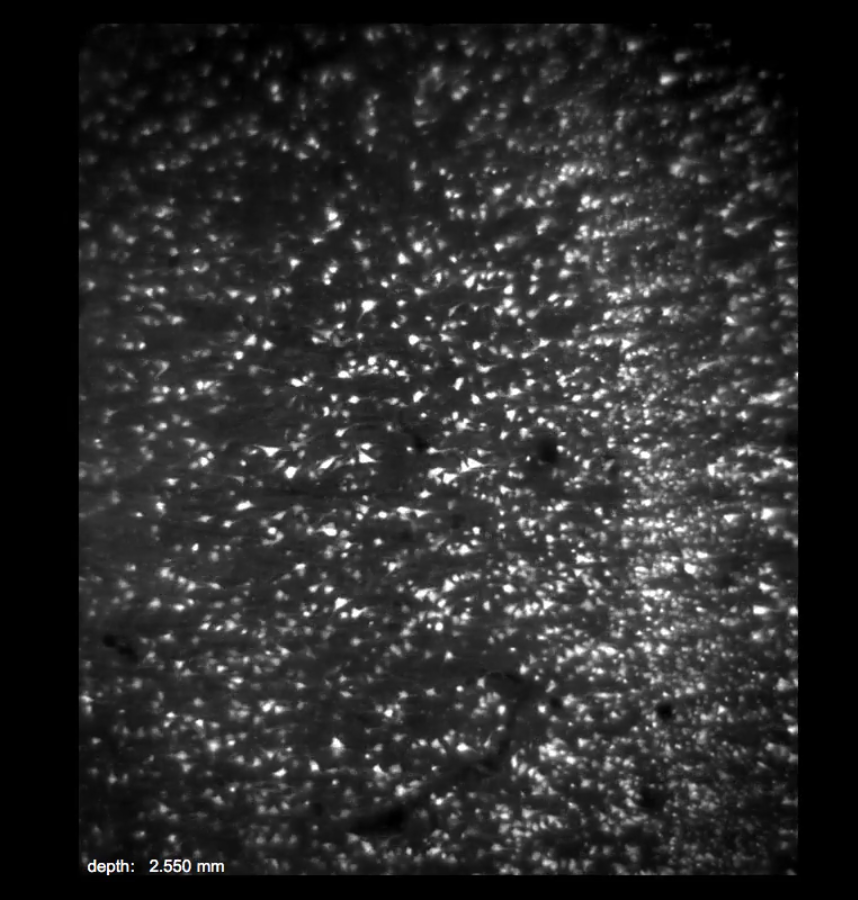


Video S5. A 2.2 mm x 2.6 mm x 5.1 mm stack of post mortem human cortex subsample (2A) stained for neurons (HuC/D) and recorded with the light sheet microscope. Pyramidal and granular cells are well delineated throughout the entire imaging volume. Signal-To-Background ratios for images obtained in superficial, middle and deep slices were 8.6 (depth 400 μm), 4.2 (depth 2600 μm) and 3.4 (depth 5000 μm) respectively.
